# Systematic review of the introduction and evaluation of magnetic augmentation of the lower oesophageal sphincter for gastro‐oesophageal reflux disease

**DOI:** 10.1002/bjs.11391

**Published:** 2019-12-04

**Authors:** E. N. Kirkham, B. G. Main, K. J. B. Jones, J. M. Blazeby, N. S. Blencowe

**Affiliations:** ^1^ Conformité Européenne Gloucestershire Hospitals NHS Foundation Trust Gloucester UK; ^2^ Bristol Centre for Surgical Research, Population Health Sciences, Bristol Medical School Bristol UK; ^3^ National Institute for Health Research Bristol Biomedical Research Centre Bristol UK; ^4^ Conformité Européenne University Hospitals Bristol NHS Foundation Trust Bristol UK

## Abstract

**Background:**

Magnetic sphincter augmentation (MSA) is reported to be an innovative alternative to antireflux surgery for patients with gastro‐oesophageal reflux disease. Although used in practice, little is known about how it has been evaluated. This study aimed to systematically summarize and appraise the reporting of MSA and its introduction into clinical practice, in the context of guidelines (such as IDEAL) for evaluating innovative surgical devices.

**Methods:**

Systematic searches were used to identify all published studies reporting MSA insertion. Data collected included patient selection, governance arrangements, surgeon expertise, technique description and outcome reporting.

**Results:**

Searches identified 587 abstracts; 39 full‐text papers were included (1 RCT 5 cohort, 3 case–control, 25 case series, 5 case reports). Twenty‐one followed US Food and Drug Administration eligibility criteria for MSA insertion. Twenty‐six documented that ethical approval was obtained. Two reported that participating surgeons received training in MSA; 18 provided information about how MSA insertion was performed, although techniques varied between studies. Follow‐up ranged from 4 weeks to 5 years; in 14 studies, it was less than 1 year.

**Conclusion:**

Most studies on MSA lacked information about patient selection, governance, expertise, techniques and outcomes, or varied between studies. Currently, MSA is being used despite a lack of robust evidence for its effectiveness.

## Graphical abstract

This systematic review summarizes published evidence for a novel procedure, magnetic sphincter augmentation of the oesophagus, a treatment for gastro‐oesophageal reflux. It found limited governance arrangements, a lack of long‐term follow‐up and varied methods of device insertion. There is a need for improvement in the rigour with which innovative surgical procedures are evaluated. Ideally, this would comprise high‐quality RCTs and long‐term registries, to allow safe adoption of the device into clinical practice.

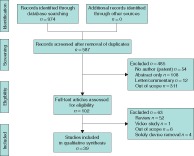

Innovation in need for IDEAL investigation

## Abstract (es)

**Antecedentes:**

El aumento de esfínter con un dispositivo magnético (*magnetic sphincter augmentation*, MSA) se ha descrito como una alternativa innovadora a la cirugía antirreflujo para pacientes con enfermedad por reflujo gastroesofágico. Aunque este procedimiento se utiliza en la práctica, se sabe poco acerca de cómo ha sido evaluado. Este estudio se propuso resumir sistemáticamente y evaluar los trabajos sobre MSA y su introducción en la práctica clínica, en el contexto de las guías (como IDEAL) para la evaluación de dispositivos quirúrgicos innovadores.

**Métodos:**

Se identificaron todos los estudios publicados que describían la colocación de MSA efectuando búsquedas sistemáticas. Los datos recogidos incluían la selección de los pacientes, disposiciones de gobernanza, experiencia del cirujano, descripción técnica, y descripción de resultados.

**Resultados:**

Las búsquedas identificaron 587 resúmenes, incluyéndose 39 artículos completos (5 estudios de cohortes, 3 estudios de casos y controles, 26 series de casos, 5 casos clínicos). En 21 estudios se siguieron los criterios de elegibilidad de la FDA para la colocación de MSA. En 26 estudios se confirmaba que se había obtenido la aprobación ética. Dos estudios describieron que los cirujanos participantes habían recibido formación en MSA; 18 proporcionaron información sobre cómo se realizó la colocación de MSA, aunque las técnicas variaron entre los estudios. El seguimiento oscilaba entre 4 semanas y 5 años; en 14 estudios fue inferior a un año.

**Conclusión:**

La mayoría de los estudios sobre MSA fueron casos aislados y series de casos, sin un incremento apreciable en la calidad de la evidencia sobre MSA. La información sobre la selección de los pacientes, gobernanza, experiencia, técnicas, y resultados estaba ausente o variaba entre los estudios, haciendo difíciles las comparaciones. En la actualidad, MSA se utiliza a pesar de la falta de evidencia robusta sobre su efectividad.

## Introduction

Gastro‐oesophageal reflux disease (GORD) is a common condition, affecting 10–20 per cent of the Western population^1^. It is associated with sequelae including Barrett's metaplasia and oesophageal adenocarcinoma, and can have a detrimental effect on quality of life^1^. The primary treatment is proton pump inhibitors (PPIs), which are generally well tolerated^1^, ^2^. Some patients, however, have recalcitrant symptoms despite treatment and others cannot tolerate, or do not wish to take, long‐term medication^2^, ^3^. In these scenarios, antireflux surgery may be offered^2^, ^3^. The most common surgical option is fundoplication, which can be associated with short‐ and long‐term complications. There is also a risk of recurrent reflux; as many as 30 per cent of patients resume PPIs within 5 years of surgery^4^. Innovative approaches to the management of refractive or recurrent reflux have therefore been sought, and one recent development is magnetic sphincter augmentation (MSA) of the lower oesophagus^5^.

First described in 2008, MSA comprises an expandable chain of magnetic titanium beads designed to augment the lower oesophageal sphincter and prevent inappropriate relaxation^5^. MSA is reported to be a safe, effective and relatively straightforward alternative to antireflux surgery^6^, ^7^, ^8^, ^9^. The LINX™ system (Torax Medical, North Shoreview, Minnesota, USA; bought by Ethicon, Johnson & Johnson, Somerville, New Jersey, USA, in March 2017) is currently the only device marketed, receiving Conformité Européenne (CE) marking in 2008, and Food and Drug Administration (FDA) approval in 2012 for use in patients fulfilling specific criteria^10^. The National Institute for Health and Care Excellence (NICE) issued guidance in September 2012 permitting the use of MSA solely under ‘special arrangements for clinical governance, consent and audit or research’ owing to ‘limited evidence of the safety and efficacy’^11^. Unlike pharmaceuticals, there is no requirement for novel devices such as MSA to be evaluated within the context of RCTs before they are marketed^12^, meaning that it can be difficult to monitor outcomes and risking delayed detection of long‐term adverse events.

A change in UK law around consent stipulates that, when patients are offered new procedures or devices, there is a requirement to provide information about the proposed treatment and all potential alternatives so they can make informed decisions^13^. This information should be based on the best available evidence, ideally from well designed and conducted RCTs. However, if devices are marketed on a background of poor‐quality evidence about the potential benefits and risks, without consistent and transparent reporting of outcomes, it may be difficult for surgeons to provide information, decide whether to offer the treatment to patients and, crucially, to obtain fully informed consent. This raises important issues about how best to evaluate surgical innovation.

The IDEAL (Idea, Development, Exploration, Assessment, Long‐term follow‐up) framework aims to overcome this problem using a stepwise means of introducing and evaluating innovative surgical procedures and devices (IDEAL‐D), with the objective of improving transparency, evaluation and reporting of innovation in surgery, and informing evidence‐based practice^12^, ^14^. Key IDEAL recommendations relate to incremental changes in study design, clinical indications, technique standardization, governance arrangements and outcomes, as the innovation progresses from first‐in‐human to long‐term follow‐up. To date, reviews of the evidence for MSA have not studied its introduction into clinical practice, so compliance with these IDEAL guidelines for evaluating innovative surgical procedures and devices is unknown^14^. This study therefore aimed to summarize and appraise the reporting of studies of MSA, to understand how this innovative procedure has been introduced and evaluated in relation to the IDEAL recommendations.

## Methods

A systematic review was undertaken to identify all published studies reporting MSA insertion. The review was conducted in line with the PRISMA statement^15^. Methods were based on those described previously^14^.

### Search strategy and study selection

Searches were undertaken in MEDLINE, Embase, CINAHL (Cumulative Index to Nursing and Allied Health Literature), the Cochrane Library, Web of Science and BIOSIS databases, from inception to January 2019. Searches consisted of subject headings and text words, combining terms for ‘magnetic sphincter augmentation’ with ‘gastro‐oesophageal reflux disease’ using the Boolean operator ‘AND’ (*Table* *S1*, supporting information).

### Study eligibility

Searches were limited to studies in humans, written in English. All primary research study designs (such as case reports, case series and comparative studies) were eligible for inclusion. Where systematic reviews were identified, reference lists were cross‐checked to ensure that all eligible studies were included. Presentations and conference abstracts were excluded because of the high probability of incomplete data. Studies reporting solely on device removal were excluded. Search results were deduplicated.

### Identification and selection of papers

Titles and abstracts were screened independently by two authors. The full‐text versions of papers retained after title and abstract screening were assessed further for eligibility. Disagreements were first discussed between the reviewers, and any unresolved conflicts referred to the wider study team. Reference lists of included papers were searched manually for additional relevant articles. Data from full‐text papers were extracted independently by at least two assessors.

### Data collection

Data collection was based on IDEAL recommendations^14^, and included information about general study characteristics, patient selection, regulatory and governance arrangements, operator and centre expertise, technique description and outcome reporting^14^. Outcome data were extracted from papers reporting follow‐up of initial studies included in the review to acquire information about long‐term outcomes; however, other data were not included to avoid double‐counting of results.

#### *General study characteristics*

The study design, year and journal of publication, country of origin, and number of participating centres and patients, were extracted. The timing of publication of studies in relation to FDA approval was also recorded. If studies involved a comparator group, eligibility criteria were compared with those for MSA, including any matching at baseline or statistical analyses to account for differences. Risk‐of‐bias assessments were undertaken for RCTs^16^.

#### *Patient selection*

Study‐specific inclusion and exclusion criteria for undergoing MSA insertion were documented, and compared with the criteria approved by the FDA in 2012. If different criteria were used, these were recorded along with any rationale. Information regarding those who were eligible to undergo MSA insertion, but did not have the procedure, was also collected.

#### *Regulatory and governance arrangements*

Reporting of information about governance approvals (for example, ethics committees, Institutional Review Boards (IRBs) and clinical effectiveness committees) was documented. Articles were assessed for whether patients were informed specifically about the innovative nature of MSA. Details of any funding from the manufacturer or other potential conflicts of interest were also noted.

#### *Operator and centre expertise*

Details of the types of centre and number of surgeons undertaking MSA insertion were recorded. Reports of surgeon and team experience with the new procedure, including any details of the learning curve and how it was accounted for, were extracted.

#### *Technique description*

All descriptions of the techniques used to insert MSA were extracted verbatim and assessed using a typology, which allows systematic deconstruction of an intervention into its individual components and steps^17^, ^18^. Descriptions of each component (such as incisions, dissection, device insertion and reconstruction) were tabulated chronologically, to identify whether: there was clear reporting of what had been performed; modifications occurred over time; and the technique had stabilized.

#### *Outcome selection, measurement and reporting*

All outcomes were extracted and categorized into groups: clinical (a clinician's or researcher's assessment of symptoms or signs)^19^; patient‐reported (a report of the status of a patient's health condition that comes directly from the patient, without interpretation of the patient's response by a clinician or anyone else)^20^; process (the specific steps that lead to a particular outcome)^21^; cost and other economic; and adverse events (an untoward medical occurrence as a result of the use of the device)^22^. The rationale for device removal and techniques used when removal was required were also documented.

In addition to recording details of reporting, adverse event and process data were summarized by calculating the range of rates for each outcome.

### Data synthesis

Results were summarized in a narrative synthesis, with descriptive statistics where appropriate. Comparisons were made between studies undertaken before and after marketing approval to establish compliance with FDA‐approved criteria. Because the study did not aim to draw conclusions about the effectiveness of MSA over other treatments, meta‐analyses were not performed.

## Results

### Characteristics of included studies

**Figure 1:**
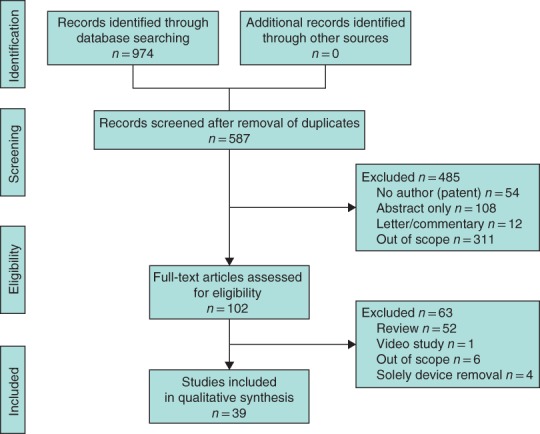
PRISMA diagram showing selection of articles for review Four papers reported longer‐term outcomes from earlier studies; this review therefore presents the results of the remaining 35 papers in detail.

**Figure 2:**
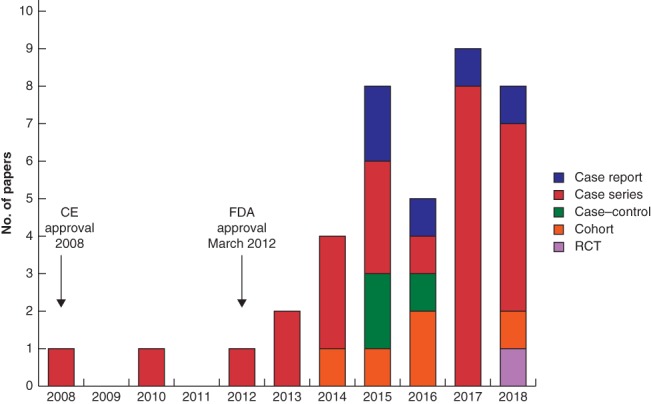
Timeline of publication year and study type relative to marketing approval CE, Conformité Européenne; FDA, Food and Drug Administration.

Systematic searches identified 974 papers and, after removing duplicates, 587 abstracts were screened (*Fig*. 1). A total of 39 full‐text papers^6^, ^10^, ^11^, ^23^, ^24^, ^25^, ^26^, ^27^, ^28^, ^29^, ^30^, ^31^, ^32^, ^33^, ^34^, ^35^, ^36^, ^37^, ^38^, ^39^, ^40^, ^41^, ^42^, ^43^, ^44^, ^45^, ^46^, ^47^, ^48^, ^49^, ^50^, ^51^, ^52^, ^53^, ^54^, ^55^, ^56^, ^57^, ^58^, published between 2008 and 2019, were finally included. The 39 articles consisted of one RCT, five comparative cohort studies (2 prospective and 3 retrospective), three comparative case–control studies, 25 case series (14 prospective, 11 retrospective) and five case reports (*Fig*. 2). Four papers^23^, ^24^, ^25^, ^26^ reported longer‐term outcomes from earlier studies. This review therefore presents the results of the remaining 35 papers in detail unless specified otherwise.

Two papers were published before FDA approval. The first^10^ was reported to be a feasibility study aimed at demonstrating the safety and efficacy of MSA, and the second^24^ reported 1‐ and 2‐year follow‐up of the initial study.

Seventeen studies were reported from a single centre. Thirty‐three studies included at least one author who had published two or more articles on MSA. Nine studies (1 RCT, 5 cohort and 3 case–control studies) included a comparator group (*Tables* 1 and 2), although only one was randomized. Of these, seven compared MSA with antireflux surgery, one^27^ compared different types of dissection technique for MSA, and the RCT^28^ compared MSA with PPIs. Five, including the RCT, reported differences in patient baseline demographics, including hernia size, obesity, age, DeMeester score and disease severity, which were not accounted for in analyses. Although four reported ‘matched’ controls, there were differences in demographics in three^29^, ^30^, ^31^, ^33^.

**Table 1:** Details of nine studies comparing magnetic sphincter augmentation with an alternative technique

				Eligibility criteria	
Reference	Study design	No. of patients	Comparator	MSA	Comparator
Louie *et al*.^44^	Cohort*	66 (34 MSA)	LNF	FDA criteria	FDA criteria
Sheu *et al*.^29^	Case–control*	24 (12 MSA)	LNF	Previous ARS, hiatus hernia > 2 cm, dysmotility, allergy to device, need for future MRI	n.r.
Riegler *et al*.^45^	Cohort	249 (202 MSA)	LNF	FDA criteria plus hernia > 3 cm, Barrett's and stage C or D oesophagitis	FDA criteria plus hernia > 3 cm, Barrett's and stage C or D oesophagitis
Reynolds *et al*.^32^	Case–control*	100 (50 MSA)	LNF	FDA criteria	FDA criteria
Asti *et al*.^33^	Cohort	238 (135 MSA)	LTF	FDA criteria	FDA criteria
Warren *et al*.^30^	Cohort*	415 (201 MSA)	LNF	FDA criteria	FDA criteria
Reynolds *et al*.^31^	Case–control*	119 (52 MSA)	LNF	n.r.	n.r.
Tatum *et al*.^27^	Cohort*	182 (all MSA)	Surgical technique	Patients undergoing MSA without previous surgery	Patients undergoing MSA without previous surgery
Bell *et al*.^28^	RCT	152 (50 MSA)	PPI	FDA criteria	FDA criteria

* Retrospective. MSA, magnetic sphincter augmentation; LNF, laparoscopic Nissen fundoplication; FDA, Food and Drug Administration; ARS, antireflux surgery; n.r., not reported; LTF, laparoscopic Toupet fundoplication; PPI, proton pump inhibitor.

**Table 2:** Differences in baseline characteristics in studies comparing magnetic sphincter augmentation with an alternative technique

Reference	BMI (kg/m^2^)		Age (years)		GERD‐HRQL score		DeMeester score		Hernia size (cm)	
	MSA	Comparator	MSA	Comparator	MSA	Comparator	MSA	Comparator	MSA	Comparator
Louie *et al*.^44^	27	30	54	47	n.r.	n.r.	n.r.	n.r.	1·4	1·5
Sheu *et al*.^29^	26·6	26·6	39·3	43·8	n.r.	n.r.	n.r.	n.r.	n.r.	n.r.
Riegler *et al*.^45^	25·7	26·1	46·6	52·8	6·4% severe GORD	61·7% severe GORD	n.r.	n.r.	1·6% > 3 cm	45·7% > 3 cm
Reynolds *et al*.^32^	26·4	26·7	53	54	19·7	18·8	n.r.	n.r.	1·5	1·6
Asti *et al*.^33^	23·9	25·1	44	50	21·0	19·7	31·4	37·6	2	2
Warren *et al*.^30^	32	40	54	52	21	19	34	39	n.r.	n.r.
Reynolds *et al*.^31^	26	27	53	53	17	19	n.r.	n.r.	n.r.	n.r.
Tatum *et al*.^27^	26·8	27·8	55·3	63·1	n.r.	n.r.	39·9	79·3	0·64	2
Bell *et al*.^28^	28	28	46	46	23·5	25·0	40·3	30·9	58% < 3 cm	49% < 3 cm

GERD‐HRQL, gastro‐oesophageal reflux disease – health‐related quality of life; MSA, magnetic sphincter augmentation; n.r., not reported; GORD, gastro‐oesophageal reflux disease.

The only RCT included 152 patients, randomized to receive PPI (102) or MSA (50) in a 2 : 1 ratio across 21 US centres. It concluded that patients should be considered for MSA rather than increased PPI doses; however, the overall risk of bias was unclear because no information was provided about the randomization process, allocation concealment or attrition.

### Patient selection

All studies involved adult patients aged over 18 years. Twenty‐one followed the FDA eligibility criteria for MSA insertion^10^. Seven, comprising a total of 427 patients and published after FDA approval, extended these criteria to include patients with a hiatus hernia greater than 3 cm^34^, ^35^ or previous gastrointestinal surgery^36^, ^37^, ^38^, ^39^, ^40^, to assess whether the device could be used in this population.

Three articles provided information about patients who were recruited but did not receive MSA. Reasons included: insurance requirements, patient preference, allergy to device metals, the potential future need for MRI (initially, this was a contraindication to MSA insertion^26^), or conversion to conventional antireflux surgery owing to more severe disease at operation than expected.

### Regulatory and governance arrangements

Twenty‐six studies reported that ethical approval had been obtained (IRB in 23, ethics committee in 3). Twelve studies were funded by the manufacturer, and 24 stated a conflict of interest in that some or all authors worked at the device company. Only 14 explicitly documented obtaining patient consent for study participation; a further six stated that consent was not needed given the study's retrospective nature.

One premarket study^10^ documented specific discussions with patients regarding the innovative use of MSA: ‘each patient was informed about the investigational nature of the trial and received detailed information about the study protocol’. One further paper^38^ reported that the authors ‘advised patients about the novelty of the approach’. None of the seven articles that extended the inclusion criteria off licence to patients outside the FDA‐approved guidelines reported discussing this with patients, although ethical approval was obtained for all seven.

### Operator and centre expertise

None of the studies provided any information about the surgical learning curve. Fifteen stated the number of surgeons performing MSA procedures (range 1–4). Seven reported entry criteria for participating surgeons (*Table* 3). Two studies reported that participating surgeons received training in the use of MSA. A further study reported that specific experience with MSA was required, but did not quantify what this comprised. The remaining five reported only that surgeons were required to have experience with antireflux surgery in general. None reported information about the expertise or training of the wider surgical team (nurses and anaesthetists). Although three studies stated that their hospitals were ‘high volume’ and one a ‘specialist reflux centre’^41^, no other information about caseload was provided.

**Table 3:** Statements of expertise of participating surgeons in included studies

Reference	Type of expertise described	Statement
Smith *et al*.^42^	Surgeons' previous experience	‘Over 20 years of experience treating GORD’
Ganz *et al*.^46^ Lipham *et al*.^25^		‘Experience with fundoplication’
Lipham *et al*.^47^		‘Proficiency in performing laparoscopic Nissen fundoplication and comfortable working at the oesophago‐gastric junction’
Bell *et al*.^28^		‘Trained and experienced in MSA’
Kuckelman *et al*.^39^	Training requirements	‘All surgeons had completed the training and certification process for MSA placement, including didactics, live case observations, and then proctoring on initial cases’
Louie *et al*.^48^		‘All participating study centres were required to have undergone training to implant the device and completed a minimum of five LINX™ implants’

GORD, gastro‐oesophageal reflux disease; MSA, magnetic sphincter augmentation.

### Technique description

Eighteen studies reported at least some information about the technique of MSA insertion, and a further nine cited one or more of these articles (*Table* *S2*, supporting information). Although the first published study^10^ documented the procedure in detail, no study described every component. Technical details were reported about the incisions (8 studies), hiatal dissection (18), device insertion (18) and crural repair (9). However, these descriptions were heterogeneous, using different wording and describing different anatomical structures. It was therefore difficult to determine whether the procedure or any of its components had evolved with time or whether they had stabilized.

With regard to the dissection technique, the need to identify the vagal trunk, access the retro‐oesophageal window and perform ‘minimal hiatal dissection’ was reported in seven, five and seven studies respectively, although the latter was not defined. One study^27^ aimed to undertake a comparison of ‘minimal hiatal dissection’ with ‘planned obligatory dissection’. During the study, however, this comparison was abandoned and all subsequent patients underwent obligatory hiatal dissection, with no explanation of why this occurred.

Of the studies providing details about device insertion, six reported how the sizing device should be inserted and three documented the required tightness around the oesophagus. Further information about the insertion technique varied between studies; for example, five reported the device location as between the posterior vagus and oesophagus, and two the retro‐oesophageal window. One study (100 patients)^6^ reported using a modified version of MSA, whereby the first 30 patients received a first‐generation and the rest a second‐generation device. Differences between the devices were described as the ‘use of a clasp instead of a suture to close the ring’ and a ‘laparoscopic sizing tool’ instead of a ‘colour‐coded sizing device’, although there was no further information and no rationale for the modification was provided. There was no documentation to suggest that patients were made aware of this change of device or that ethical approval was sought. No other studies documented which generation of device was used.

Eleven studies reported that crural repair was undertaken at the discretion of the surgeon, and technical information was included in nine. Of these, two described a posterior cruroplasty using ‘a permanent suture’ and ‘with one or two sutures’, whereas seven stated only that ‘posterior cruroplasty’ was undertaken. Four studies documented a rationale for crural repair: ‘…when the hiatus appeared patulous or a sliding hernia was present’ 6, 42, and ‘if hiatal hernia visible after posterior dissection of hiatus that kept the phreno‐oesophageal membrane intact’^31^, ^32^.

### Outcome selection, measurement and reporting

Outcome reporting from all 39 papers is summarized in *Table* 4. Thirty‐eight different outcomes were reported across all studies. No single outcome was measured in all studies. No study provided a rationale for the outcomes selected. Duration of follow‐up ranged from 4 weeks to 5 years; this was not reported in one study. Five‐year data were available from two studies and the duration of follow up was less than 1 year in 14.

**Table 4:** Outcome reporting in included studies

	No. of studies reporting outcomes (*n* = 39)	Range of rates reported (%)*
**Adverse events**		
Device removal	27	0·5–8·3
Device erosion	8	0·1–1·2
Need for dilatation	22	2–67
Readmission to hospital within 30 days	13	1·3–5·4
Mortality	3	0
**Clinical**		
DeMeester score	7	–
Manometry	4	–
Oesophageal pH testing	10	–
Oesophagogastroduodenoscopy	13	–
Barium swallow	7	–
Proton pump inhibitor use	25	–
**Process**		
Duration of operation (min)	16	23–184†
Duration of hospital stay (days)	13	0–3†
**Patient‐reported outcomes**		
GERD‐HRQL score	31	–
Other validated patient‐reported outcomes	7	–
Non‐validated patient‐reported outcome	5	–
**Economic**		
Costs associated with device	1	–

* Unless indicated otherwise;

† values are median (range). GERD‐HRQL, gastro‐oesophageal reflux disease – health‐related quality of life.

#### *Clinical outcomes*

Objective clinical outcomes were reported in 25 studies. These included data from DeMeester scores (7 studies), manometry (4), oesophageal pH measurements (10), oesophagogastroduodenoscopy (13) and barium swallows (7). Fifteen of the 25 studies reported obtaining more than one of these objective outcomes. These assessments were undertaken at variable times after surgery, ranging from 1 day to 3 years. Three studies performed these tests beyond 1 year after operation. The objective clinical data were infrequently correlated with the patient‐reported outcomes reported below.

#### *Patient‐reported outcomes*

The validated gastro‐oesophageal reflux disease health‐related quality of life (GERD‐HRQL) questionnaire was used in 31 studies. Baseline questionnaires were administered before operation in all 31 studies and at various other time points, ranging from 10 days to 5 years after surgery. Only five studies reported use of the questionnaire after a year. Other assessment measures included a Foregut Symptom Questionnaire (4 studies), Respiratory Index Score (1), Quality of Life in Reflux and Dyspepsia score (1) and Modified Dakkak dysphagia severity score (1). Five remaining studies reported patient‐reported outcomes, including incidence of dysphagia, change in reflux, gas‐related symptoms and symptoms of odynophagia, but did not define these or use a named questionnaire.

#### *Process outcomes*

Operating time and duration of hospital stay were reported in 16 and 13 studies, respectively.

#### *Cost and economic outcomes*

One study reported an economic outcome: overall hospital charges associated with device insertion.

#### *Adverse events*

Twenty‐two studies reported the need for postoperative oesophageal balloon dilatation for dysphagia or odynophagia, eight described cases of device erosion into the oesophagus, and 13 readmission to hospital (for reasons including dehydration, dysphagia, pain, nausea and pulmonary embolus). No intraoperative complications were reported in any paper.

#### *Device removal*

Of the 27 studies reporting the need for device removal (total 84 patients), the timing ranged from 21 days to 47 months after insertion. No prespecified criteria or rationale were provided. Reasons for device removal were reported in all studies, and included erosion, migration, development of oesophageal cancer, ongoing refractory symptoms, the need for MRI and patient preference. The surgical approach to device removal was poorly documented in these studies, and included laparoscopic, endoscopic and laparoendoscopic techniques.

## Discussion

This comprehensive review of the reporting of an innovative surgical device – MSA of the lower oesophagus – summarizes information from 39 studies published between 2008 and January 2019. Only one small RCT was identified; most studies were case studies or case series with serious shortcomings. Of the nine comparative studies, including one RCT, eight were limited by different selection criteria and unmatched patients at baseline. Information about ethical approval, patient consent and conflicts of interest was often missing. Many studies reported using MSA for indications outside the current FDA regulations. Reporting of the technical aspects of device insertion was either lacking or varied between studies, making it difficult for surgeons to replicate the technique and learn from others' experiences. Currently, therefore, MSA is being used in clinical practice despite a lack of robust evidence to support its effectiveness, potentially placing patients, surgeons and healthcare providers at risk. Although some guidance for evaluating innovative surgical procedures and devices is available (IDEAL recommendations), it was not followed. Pilot work is now required to address the aforementioned issues and enable an RCT comparing MSA with current treatments to be designed optimally, to inform decision‐making and patient care. Moreover, a registry to monitor device insertions, document long‐term outcomes including adverse events, record surgeon experience and allow safe widespread adoption of the device into clinical practice, is required.

The Royal College of Surgeons of England has urged the government to ‘act urgently to reform the lax regulation system governing medical devices, including a compulsory registry of all new implants’^12^, ^59^. Such a system would need to strike a balance between avoiding burden and encouraging the development of well founded evidence on safety and efficacy^12^, ^59^. There has been a recent call for new surgical procedures and implants to be tested in RCTs before being made routinely available, in line with IDEAL recommendations and existing pathways for introducing pharmaceutical products^12^. In the UK, the Medicines and Healthcare products Regulatory Agency^60^ regulates medical devices with the aim of ensuring that they are safe and efficacious. The type of study required to achieve approval is not specified, but the majority do not include a control group, despite the Royal College calling for more RCTs to be undertaken and encouraging surgeons to use IDEAL guidance^59^. In the USA, invasive devices are now subject to a pivotal clinical study as part of the FDA's premarket approval pathway^12^. Although MSA did undergo a pivotal study, subsequent publications failed to build on its findings and long‐term outcomes have not been established, particularly when compared with current treatments (PPIs and antireflux surgery).

This is concerning in the context of recent high‐profile devices that have later caused serious health problems despite complying with regulatory processes. One such example is transvaginal mesh, which is no longer offered routinely as the result of an independent review^61^. Another example is robotic surgery. Although this has been introduced rigorously in some surgical disciplines (robotic prostatectomy has been endorsed by NICE for centres performing more than 150 procedures per year), such evaluation has not been applied uniformly. Conversely, a recent review^62^ conceded that, despite a lack of evidence to support any benefit, cardiac surgery ‘represents one of the largest markets in the field of robotic surgery’, and the first robotic mitral valve replacement in the UK resulted in the death of the patient^63^. Following this, guidance regarding the clinical governance, oversight and infrastructure required when introducing new innovations has been published. The guidance reinforces the responsibilities of surgeons and surgical societies in arranging appropriate training and mentorship, and submission of data to national registries, thereby placing patient safety at the core^64^. Another example of a device that was introduced before full evaluation – and subsequently removed from practice – is the Angelchik prosthesis. Similar to MSA, it gained popularity as a solution to GORD in the 1980s because of its technical simplicity^65^. Although data from initial non‐randomized studies were promising, an RCT was stopped prematurely owing to adverse events, and long‐term follow‐up detected erosions and migrations necessitating reoperation in 25 per cent of patients^66^. As a result, use of the device was almost entirely abandoned by the early 2000s, by which time over 25 000 had already been inserted.

Despite this, MSA continues to be used across the world and over 3000 device insertions were undertaken in the USA between 2012 and 2016^43^. A registry that was funded and sponsored by the device manufacturer existed until 2016. However, this did not include all patients and follow‐up was limited to 3 years because it closed prematurely for unknown reasons. Without registries to monitor outcomes of new devices, there can be delays in understanding the short‐ and long‐term risks of new devices and procedures^67^.

This study has tracked the introduction and evolution of MSA from the first published description to the present day. Despite this, however, there are limitations. Outcomes were not analysed in depth and meta‐analyses were not undertaken. Exclusion of non‐English language papers may mean that important additional findings were missed. Data were extracted verbatim, and the authors assumed that if something was not documented, it did not happen; authors were not contacted individually for further information. It is also possible that the device is used much more widely and other cases have not been published. A final limitation is that one paper was published before IDEAL was introduced in 2009 (and therefore could not have followed these guidelines rigorously), and a further 16 were published before publication of the updated IDEAL‐D framework specific for surgical devices in 2016. More work is needed to establish why the IDEAL guidance has not been followed; despite this, the fundamental principles of IDEAL are not new concepts and form the basis of good research.

This in‐depth analysis of reporting of an innovative invasive procedure corroborates existing evidence^12^ that surgical procedures are often poorly evaluated before implementation in clinical practice. The long‐term safety of MSA has not been sufficiently demonstrated in the existing 39 published studies owing to inconsistent reporting of outcomes, particularly those detailing long‐term follow‐up. There is a lack of standardized, transparent reporting of how the MSA device should optimally be inserted, making adoption into practice difficult and hampering comparisons between studies. There is a need for robust assessment and reporting to improve the rigour with which innovative surgical procedures are evaluated, to optimize transparency, maximize patient benefit and reduce harms.

## Editor's comments

What have we learned from the past? Not much, according to this study by Kirkham *et al*. The surgical community falls short in the proper evaluation of new surgical devices before implementation in clinical practice. We have to do better in terms of informing patients on experimental treatments and being critical on new technologies. On the other hand, we want to make surgery safer, more accessible and improve the outcomes, and technological improvements are needed to achieve these goals. However, I share the authors' conclusion that ‘magnetic sphincter augmentation is being used in clinical practice despite a lack of robust evidence to support its effectiveness, placing surgeons and patients at risk’. There is a clear need for international registries that evaluate indications and outcomes of new devices and techniques. National surgical bodies and governments should support these registries so they are independent of the industry/companies and surgeons involved in the manufacturing and promotion of new devices, because there will always be a conflict of interest.

B. P. L. Wijnhoven

*Editor, BJS*

## Supporting information


**Table S1.** Ovid MEDLINE search strategy
**Table S2.** Descriptions of the key components of MSA insertion
