# Supplementary material for: Systematic review of the introduction and evaluation of magnetic augmentation of the lower oesophageal sphincter for gastro‐oesophageal reflux disease
Source: Br J Surg. 2019 Dec 4;107(1):44–55. doi: 10.1002/bjs.11391 (PMC6972716; doi:10.1002/bjs.11391)
Supplement: Supplementary file 1 — Table S1. Ovid MEDLINE search strategy Table S2. Descriptions of the key components of MSA insertion [file BJS-107-44-s001.docx]

**BJS11391**

**Systematic review of the introduction and evaluation of magnetic augmentation of the lower oesophageal sphincter for gastro-oesophageal reflux disease**

E. N. Kirkham, B. G. Main, K. J. B. Jones, J. M. Blazeby and N. S. Blencowe

**Table S1 Ovid MEDLINE search strategy**

|  | **Search terms** |
| --- | --- |
| 1 | Esophagus / or esophagus epithelium / or esophagus mucosa / or esophagus muscle / or gastroesophageal junction / or lower esophagus sphincter / or upper esophagus sphincter |
| 2 | Gastroesophagealreflux / or non erosive reflux disease / or reflux esophagitis / or esophageal mucosa / or oesophageal sphincter, upper / or esophagogastric junction / or esophageal sphincter, lower |
| 3 | Oesophagi*.ti,ab,kf |
| 4 | Esophag*.ti,ab,kf |
| 5 | Gastro-oesophageal.ti,ab,kf |
| 6 | Gastroesophageal.ti,ab,kf |
| 7 | Gasto-esophageal.ti,ab,kf |
| 8 | Reflux.ti,ab,kf |
| 9 | GERD.ti,ab,kf |
| 10 | GORD.ti,ab,kf |
| 11 | 1 or 2 or 3 or 4 or 5 or 6 or 7 or 8 or 9 or 10 |
| 12 | Linx.ti,ab,kf |
| 13 | Magnetic sphincter augmentation.ti,ab,kf |
| 14 | MSA.ti,ab,kf |
| 15 | (augment* adj3 sphincter).ti,ab,kf |
| 16 | (prevent* adj3 relax).ti,ab,kf |
| 17 | Sphincter device*.ti,ab,kf |
| 18 | (beads adj4 laparoscop*).ti,ab,kf |
| 19 | Dynamic implant&.ti,ab,kf |
| 20 | 12 or 13 or 14 or 15 or 16 or 17 or 18 or 19 |
| 21 | 11 and 20 |
| 22 | Exp animals / not humans.sh |
| 23 | 21 not 22 |
| 24 | Exp animal / or non human/ |
| 25 | Exp human/ |
| 26 | 24 not 25 |
| 27 | 21 not 26 |
| 28 | 23 or 27 |
| 29 | Remove duplicates from 28 |

**Table S2 Descriptions of the key components of MSA insertion**

| **Ref** | **Incisions** | **Dissection for device insertion** | **Device Insertion** | **Crural repair** |
| --- | --- | --- | --- | --- |
| 10 | 11mm, 12mm and 3x 5mm ports | Peritoneal reflection anterior to the gastroesophageal junction was divided to expose the esophageal wall…The retro-esophageal dissection began along the border of the right crus…the posterior vagal trunk was identified. The same dissection was repeated along the left crus. Gentle dissection from the right opened the retro-esophageal window | Sizing tool advanced through the posterior esophageal tunnel, and wrapped around the esophagus. Device selected by alignment of the white bead with one of the coloured beads. Sizing tool removed and MSA device inserted. Sutures at both end…secured with a Ti-Knot. | NR |
| 24 | NR | Visceral peritoneum on the anterior surface of the esophagogastric junction is divided to expose the anterior esophageal wall… Gentle dissection from the right opens the retroesophageal window, and a tunnel is created between the posterior esophageal wall and the posterior vagal trunk | Sizing tool is passed through the posterior esophageal tunnel and wrapped around the esophagus. LINX passed through the tunnel, wrapped around the esophagus, laid in the incision created in the visceral peritoneum over the anterior surface of the GEJ. The sutures at both ends of the device are secured with a Ti-Knot | Posterior cruroplasty |
| 25 | NR | Begins along the anterior border of the right crus just cephalad to the decussation of the crura. The posterior vagal trunk is identiﬁed. The same dissection is repeated along the left crus of the diaphragm. Gentle dissection from the right opens the retroesophageal window, and a tunnel is created | A sizing tool is advanced through the tunnel and wrapped around the tubular esophagus…Appropriate sized device is inserted and ends secured | NR |
| 6 | NR | Minimal dissection to create the space where the device would encircle the LES when implanted | Sizing instrument used to determine the circumferential diameter of the oesophagus at the GEJ…appropriately sized device is placed through the opening of the vagus nerve and oesophagus. The ends of the device are…secured | Posterior crural repair |
| 41 | NR | NR | Size was measured via a sizing tool and the appropriate magnetic ring was wrapped around the lower oesophageal sphincter | NR |
| 49 | NR | The GEJ was minimally dissected from the right and left crus of the diaphragm to create a space for the device. The hepatic branch of the vagus nerve was preserved | Laparoscopic sizing device was placed around the esophagus and adjusted until snug to determine the size of the LINX device. Device placed around oesophagus and secured using a Ti-Knot system or clasp device | Re-approximation of the crura |
| 42 | NR | Minimal dissection technique to create space | NR | Posterior crural stitch |
| 44 | Similar to LNF | Landing zone created by incising the peritoneum lateral left crus and posterior fundus…tunnel created behind the oesophagus…on anterior surface, peritoneum and fat were cleared with cautery | Outer circumference of oesophagus measured using a sizing device. Tightened until it approximates the circumference of the oesophagus without indenting the tissue. Device situated in tunnel between posterior vagus and oesophagus… and sutures secured using a suture-securing device | NR |
| 32 | 2x5mm, 8mm, Nathonson’s | The hepatic branch of the vagus nerve is identiﬁed and preserved. The right and left crura are identiﬁed and minimally dissected to create a tunnel behind the esophagus | The provided sizing device is used to determine the number of beads on the device. MSA device pulled through retro-oesophageal window…secured using clasp | NR |
| 29 | NR | Limited dissection of the phrenoesophageal ligament to allow development of a retroesophageal window at the decussation of the right and left crus. The posterior vagus was identiﬁed, a window was created between the posterior vagus and esophagus | Sized according to the manufacturer’s instructions, and the device was subsequently placed to encircle the esophagus, tied with a Ti-Knot device | NR |
| 30 | Similar to LNF | Minimal hiatal dissection, and preservation of the gastroesophageal junction, speciﬁcally the phrenoesophageal ligament, and gastric anatomy | NR | Posterior closure of the crura with 1–2 sutures |
| 31 | 2x5mm ports, 8mm port, Nathonson’s | The hepatic branch of the vagus nerve is identiﬁed and preserved. The right and left crura are identiﬁed and minimally dissected to create a tunnel behind the esophagus | The provided sizing device is used to determine the number of beads on the device. MSA pulled through the retro-oesophageal tunnel, secured using clasp | NR |
| 33 | NR | Peritoneal reflection overlying the oesophago-gastric junction was divided | Special sizing instrument used to measure the circumference of the oesophagus and an appropriately sized device placed | NR |
| 38 | The same as sleeve gastrectomy | Lysis of adhesions…crura of diaphragm exposed | Sizer placed between posterior vagus nerve and oesophagus…repeated several times to assure accuracy. LINX placed anterior to posterior vagus nerve and buckled in place | NR |
| 34 | NR | By ﬁrst identifying the right crus and developing a plane between the crus and the hernia sac | The esophagus was measured at the gastroesophageal junction using a sizing tool and the appropriate LINX device was implanted | Primary posterior cruroplasty |
| 35 | NR | Routine dissection of the hernia sac and mediastinum until adequate oesophageal length obtained | The MSA sizer was introduced until it rested smoothly but non compressively against the non-distended oesophagus and an MSA with the corresponding beads was placed between posterior oesophagus and posterior vagus…clasp actuated | Performed with permanent suture |
| 11 | As per LNF | Minimal dissection with partial division of the phrenooesophageal ligament to enable access to the distal oesophagus | Application of the LINX sizing device, insertion and locking of LINX device | Crural repair |
| 50 | NR | Complete hiatal dissection with reduction and resection of the hernia sac from the mediastinum, mobilization of the thoracic esophagus to provide 2–3 cm of intra-abdominal esophageal length” | External circumference of the gastroesophageal junction was measured and an appropriately sized LINX device placed | Primary posterior cruroplasty |
| 58 | NR | NR | Adequate ring size was measured with the sizing tool and the magnetic ring was wrapped around the lower end of the lower esophageal sphincter | Not reported |
| 40 | 5 ports | Upper abdominal adhesiolysis | Tunnel between oesophagus and posterior vagus nerve and a 16-bead MSA was inserted and locked | Standard posterior crural repair |
| 27 | NR | NR | NR | Posterior stitch cruroplasty |

Key: MSA = Magnetic Sphincter Augmentation, LNF = laparoscopic Nissen fundoplication, LES = lower oesophageal sphincter, GEJ – gastro-oesophageal junction, NR = not reported
